# Cyberbullying Victimization, Self-Esteem and Suicidal Ideation in Adolescence: Does Emotional Intelligence Play a Buffering Role?

**DOI:** 10.3389/fpsyg.2018.00367

**Published:** 2018-03-22

**Authors:** Natalio Extremera, Cirenia Quintana-Orts, Sergio Mérida-López, Lourdes Rey

**Affiliations:** ^1^Department of Social Psychology, Universidad de Málaga, Málaga, Spain; ^2^Department of Personality, Evaluation and Psychological Treatment, Universidad de Málaga, Málaga, Spain

**Keywords:** cybervictimization, adolescence, emotional intelligence, suicide ideation, self-esteem

## Abstract

Cyberbullying has been linked to social, physical and psychological problems for adolescent victims but there has been no analysis of the specific role of emotional intelligence in protecting against the negative symptoms associated with cyberbullying victimization. This study examined the interaction between cyberbullying victimization and emotional intelligence (EI) as predictors of psychological maladjustment (operationalized as high suicidal ideation and low self-esteem) in 1,660 Spanish adolescents. We also investigated whether levels of EI moderated the relationship between cyberbullying victimization and mental health problems. The cyberbullying victimization x EI interaction contributed to variance in suicidal ideation and self-esteem in our sample of adolescent victims. Adolescent victims of cyberbullying with high EI scores reported lower suicidal ideation and higher self-esteem than their less emotionally intelligent counterparts. Thus, our data provide empirical support for theoretical and conceptual work connecting victimization, EI abilities and mental health associated with cyberbullying. This suggests that alone, but also in combination, EI may be particularly relevant in leading to increased levels of mental health issues in cyberbullying victims. Finally, the theoretical implications of our findings on the relationship between these variables and the mental health issues of adolescent victims of cyberbullying are discussed.

## Introduction

Internet and electronic devices have given adolescents novel ways of socializing but have also made possible new kinds of negative interactions, known as cyberbullying (Tokunaga, 2010; Palermiti et al., 2017). Although various definitions of cyberbullying have been proposed, it is commonly defined as “an aggressive, intentional act carried out by a group or individual, using electronic forms of contact, repeatedly and over time against a victim who cannot easily defend him or her-self” (Smith et al., 2008, p.376). In particular, cyberbullying involves harassing, intimidating, threatening or otherwise harming others by sending or posting threatening or humiliating texts, pictures or videos over the Internet without permission (Patchin and Hinduja, 2010). Compared with traditional bullying, the relationship between perpetrator and adolescent victim is more complex, due to the anonymity that is possible on electronic media, as well as the rapid social dissemination, lack of supervision, permanence of the material and the easy access that bullies have to their victims (Juvonen and Gross, 2008; Della Cioppa et al., 2015). Globally, a significant proportion of young people are victims of cyberbullying, with prevalence ranging from as low as 6.5 or 10% (Ybarra and Mitchell, 2004; Mishna et al., 2011) to as high as 72% (Juvonen and Gross, 2008). However, there seems to exist some gender differences with regard to prevalence, with higher victimization rates among girls in 70% of the countries and showing decline with age in about two thirds of the countries (Craig et al., 2009).

Although the psychological consequences of cyberbullying appear to be very similar to those of traditional bullying, being a victim of cyberbullying has a greater negative effect on adolescents' psychosocial adjustment than traditional bullying (Mishna et al., 2011; Campbell et al., 2012). Victims of cyberbullying report more social and emotional problems, such as isolation and emotion regulation problems, than victims of traditional bullying (Ak et al., 2015; Elipe et al., 2015). Research has also shown that cyberbullying has negative consequences for both the physical health and psychological adjustment of victims (Tsaousis, 2016). Young people who were victims of cyberbullying reported higher levels of sleep problems and bed wetting than their non-victimized peers (Monks et al., 2009). Student victims of cyberbullying are at increased risk of depression, anxiety and substance abuse (Takizawa et al., 2014; Palermiti et al., 2017).

Cyberbullying also has a negative effect on victims' self-esteem (Patchin and Hinduja, 2010; O'Brien and Moules, 2013). Self-esteem, often defined as “a favorable or unfavorable attitude toward the self” (Rosenberg, 1965, p.15), is critical during adolescence, when identity development is heavily influenced by one's social relationships (Leary and Downs, 1995). Scholars have shown that a low self-esteem, among other relevant variables, is both a strong predictor of cyberbullying victimization and also a negative consequence after a cyberbullying experience (Egan and Perry, 1998). Recent studies investigating the relationship between cyberbullying and self-esteem have found that victims of cyberbullying report lower self-esteem (Chang et al., 2013; Cénat et al., 2014). While the causal link is not clear, some authors suggest that victims of bullies who use the Internet to harass and mock may be more likely to develop low self-esteem which, in turn, can have severe consequences for young people's well-being and psychological adjustment (Palermiti et al., 2017), including increasing the risk of suicide. Suicide is the second most frequent cause of death amongst those aged between 15 and 29 years old (World Health Organization, 2014). Although few studies have investigated the relationship between cyberbullying and suicide, existing results suggest that the risks of suicidal behavior and suicidal ideation are higher in those who have been victimized (Hinduja and Patchin, 2010; van Geel et al., 2014). If cyberbullying is not handled appropriately, it may led to negative emotional responses and poor psychological adjustment (Ortega et al., 2012). Cyberbullying has a negative effect on adolescent development and is typically associated with multiple psychological problems. Some researchers have begun to study personal resources that facilitate coping with cyberaggression by peers and are therefore protective (Chen et al., 2017). These personal resources might ameliorate the potentially negative effects of cyberbullying and may include emotional intelligence (EI).

EI is defined as a set of cognitive-emotional skills for processing emotional information in order to promote emotional and intellectual growth (Mayer and Salovey, 1997). EI encompasses the ability to perceive and express emotion, the ability to use emotional information to facilitate thought, the ability to use emotional reasoning and understanding and the ability to regulate emotions (Mayer et al., 2008). Research has shown that people high in EI are more likely to be aware of their own emotions and to express and regulate emotions more effectively than their lower EI peers and that they also have lower rates of psychopathologies related to emotional difficulties (for a review see Mayer et al., 2008; Martins et al., 2010; Resurrección et al., 2014). An emerging body of research shows that EI seems to play a key role in psychological adjustment in adolescence (Baroncelli and Ciucci, 2014; Resurrección et al., 2014). Several studies have shown that adolescents with greater EI are more likely to experience positive social relationships and better psychological adjustment (e.g., better coping strategies, fewer internalizing problems) than their lower EI peers (Lomas et al., 2012; Baroncelli and Ciucci, 2014), so EI is a potential target for school-based interventions aimed at weakening the link between cyberbullying victimization and psychological maladjustment during adolescence.

To date, there has been relatively little research directly investigating whether EI helps to mitigate the negative consequences associated with cyberbullying. One study found that adolescents with low IE were more likely to be victims of cyberbullying than their higher EI peers; in other words, adolescents who are poor at perceiving, expressing and regulating emotions are more likely to be cyberbullied (Baroncelli and Ciucci, 2014). Research has also pointed out EI as an important protective factor against the negative consequences of cyberbullying victimization as it might buffer mental health problems by promoting positive ways of coping (e.g., Davis and Humphrey, 2012b). Indeed, earlier studies have found that, in university students, EI skills moderate the relationship between cyberbullying victimization and its emotional impact, suggesting that developing emotional skills might be a way of minimizing the negative consequences of victimization (Elipe et al., 2015). Finally, Davis and Humphrey (2012a) found that, in a sample of adolescents, EI moderated the relationship between various negative life stressors and mental health. These authors have also highlighted that pathways linking EI to better mental health are complex and suggested further research on those pathways to the prediction, understanding and attenuation of maladjustment in youth. In this case, despite the increasing interest in the association between EI and psychological problems during adolescence, the role of EI as a potential buffer against the influence of cyberbullying on psychological problems has not yet been examined. This study addresses this gap.

### This study

The aim of this study was to provide further evidence on the potential moderation of the relationship between cyberbullying victimization and psychological adjustment by EI in adolescence. Three specific objectives were defined. First, to explore the relationship between cyberbullying victimization, EI, self-esteem and suicide risk in a relatively large sample of adolescents in order to extend our understanding of the correlates of cyberbullying victimization experiences in adolescence. Second, to test whether sociodemographic factors (i.e., gender, age and grade levels) and EI showed differential patterns in predicting our main variables (i.e., cyberbullying victimization, self-esteem, and suicide risk). Finally, to investigate EI as a potential moderator of the relationship between cyberbullying victimization and positive (e.g., self-esteem) and negative (e.g., suicide risk) psychological outcome variables.

Based on previous research, we expected to find cyberbullying victimization to be positively related to suicide risk and negatively associated with self-esteem, whereas we expected to find EI to be negatively related to suicide risk and positively associated with self-esteem. Furthermore, as an important and positive factor associated with psychological adjustment (e.g., Davis and Humphrey, 2012b), we hypothesize that EI might serve as a buffer between cyberbullying victimization and psychological maladjustment in adolescents. More specifically, we hypothesize that those adolescents with higher levels of EI would report lower levels of suicide risk and greater self-esteem.

## Materials and methods

### Participants

The sample consisted of 1,660 adolescents (50.4% female) studying in six public high schools in Málaga (Andalusia, Spain). The mean age was 14.10 years (*SD* = 1.54; range 12–18). Participation in the study was voluntary and confidential. The study was carried out in accordance with the Declaration of Helsinki (2013) and was approved by the Research Ethics Committee of the University of Malaga (Spain).

### Procedure

As the implementation and evaluation of the research fell under the discretion of the head teachers, written consent for individual participants was provided by school authorities, who were responsible for consulting and reporting to the parents about the research and made the final decision on their research participation. No parents refused the adolescents' participation as they believed that this research for detection of potential bullying in their educational center was a beneficial initiative for school quality of life of students, parents and teachers. Therefore, the assessment was carried out in classrooms during the normal school schedule, with guarantees of the participants' voluntariness and anonymity and with the written approval of the school authorities. The questionnaires were completed during a 1-h lesson during the last *two* trimesters of the 2016/2017 academic year, with the exact time dependent on the schedule of individual schools. In addition, students, parents and school authorities were fully aware that by completing the questionnaires they were in fact providing informed consent to use this data anonymously in the present research. The questionnaires were administered to the classes in sessions with *one* of the researchers and at least *one* teacher from the school present. All participants were encouraged to provide honest answers.

#### Cyberbullying victimization

Cyberbullying victimization was measured by using the cybervictimization subscale of the European Cyberbullying Intervention Project Questionnaire (ECIP-Q; Brighi et al., 2012). The ECIP-Q is a 22-item self-report measure dealing with cyberbullying in the previous 2 months. It includes items about insults addressed directly to the respondent, insulting comments made to others about the respondent; threats; identity theft; use of the respondent's identity without permission; theft of private information; displays of private information; embarrassing videos and pictures; manipulation of pictures; social exclusion; spreading of rumors (Elipe et al., 2015). The cybervictimization subscale comprises 11 items to which responses are given using a 5-point Likert scale ranging from 0 (never) to 4 (more than once a week). The scale was used in the Spanish version by Ortega-Ruiz et al. (2016), which has been reported to have adequate reliability and validity. In our sample, Cronbach's alpha for the cybervictimization subscale was 0.86.

#### Emotional intelligence

EI was measured with the Wong and Law Emotional Intelligence Scale (WLEIS; Wong and Law, 2002), which consists of 16 items, four items for each subscale: self-emotion appraisals (SEA), others' emotion appraisals (OEA), regulation of emotion (ROE), and use of emotion (UOE). Each item is rated on a seven-point Likert scale ranging from 0 (totally disagree) to 6 (totally agree). As we were interested in the overall construct, we summed the subscale scores to yield a global perceived EI score, with higher scores indicating greater EI. Previous research has supported score reliability (e.g., Mérida-López et al., 2017). In the present study, the Spanish version of the WLEIS had acceptable reliability, with the internal consistency coefficient for the total WLEIS score of 0.88 and the composite reliability of 0.83. The average variance extracted and McDonald's Ω was of 0.82 for both indexes.

#### Suicidal thoughts and behaviors

Suicidal thoughts and behaviors were assessed with the Suicidal Behaviors Questionnaire–Revised (SBQ-R; Osman et al., 2001) which provides an indication of overall suicidality. It comprises four items to which responses are given using a Likert scale: lifetime suicidal ideation and suicide attempts, frequency of suicidal ideation in the past year, communication of suicidal intent and likelihood of future suicidal behavior; higher scores indicate greater suicidality. The SBQ-R was translated from English into Spanish using back-translation. Research has supported score reliability (Extremera and Rey, 2016). In this research, the Spanish version of the SBQ-R showed acceptable reliability, with an internal consistency coefficient of 0.87 and a composite reliability of 0.93. The average variance extracted and McDonald's Ω was of 0.90 for both indexes.

#### Self-esteem

Self-esteem was measured using the Rosenberg Self-Esteem Scale (RSES; Rosenberg, 1965). The RSES is a self-report measure which was designed to assess global self-esteem. The RSES consists of 10 items, 5 positively worded and 5 negatively worded, to which responses are given using a Likert scale ranging from 1 (strongly disagree) to 4 (strongly agree). We used the Spanish version (Martín-Albo et al., 2007), for which adequate reliability and validity have been reported. In our sample the internal consistency coefficient was 0.87.

### Data analyses

First, we computed descriptive statistics and calculated Pearson's product moment correlations (two-tailed) between cyberbullying victimization (independent variable), EI (moderator), self-esteem and suicide risk (dependent variables). We used SPSS (version 22) for these analyses. Second, we carried out moderation analyses with self-esteem and suicide risk as dependent variables to explore potential moderation of the relationship between cyberbullying victimization and adolescents' psychological adjustment by EI. The SPSS PROCESS macro was used to conduct these analyses (Hayes, 2013). Standard procedures were followed, with the number of bootstrap resamples set to 5,000 with 95% confidence intervals.

## Results

### Descriptive analyses

Descriptive statistics (mean, standard deviations, and reliabilities) and bivariate correlations among the study variables are displayed in Table 1. As the table shows, there were correlations among cyberbullying victimization, EI and two indicators of psychological adjustment (self-esteem and suicide risk) in the expected directions. First, cybervictimization was negatively related to EI and self-esteem, but positively associated with suicide risk. In addition, EI was positively related to self-esteem and negatively linked to suicide risk. Lastly, self-esteem was negatively associated with suicide risk. Both the total score of EI and the subscales were linked to self-esteem and suicide risk in the expected directions. In particular, use of emotion was the dimension with the strongest link to self-esteem, whereas regulation of emotion showed the highest association with suicide risk.

**Table 1:** Descriptive statistics and intercorrelations among study variables.

	**1**	**2**	**3**	**4**	**5**	**6**	**7**	**8**
1. Cybervictimization	-							
2. Emotional Intelligence	−0.13^**^ [−0.19 to −0.07]	-						
3. SEA	−0.14^**^ [−0.20 to −0.08]	0.83^**^ [0.81 to 0.85]	-					
4. OEA	0.02 [−0.04 to 0.08]	0.63^**^ [0.59 to 0.67]	0.41^**^ [0.36 to 0.46]	-				
5. ROE	−0.15^**^ [−0.21 to −0.09]	0.81^**^ [0.79 to 0.83]	0.62^**^ [0.58 to 0.66]	0.26^**^ [0.20 to 0.32]	-			
6. UOE	−0.11^**^ [−0.17 to −0.05]	0.81^**^ [0.79 to 0.83]	0.54^**^ [0.49 to 0.58]	0.38^**^ [0.33 to 0.43]	0.54^**^ [0.49 to 0.58]	-		
7. Self-esteem	−0.22^**^ [−0.28 to −0.16]	0.49^**^ [0.44 to 0.54]	0.41^**^ [0.36 to 0.46]	0.13^**^ [−0.19 to −0.07]	0.43^**^ [0.38 to 0.48]	0.51^**^ [0.46 to 0.56]	-	
8. Suicide risk	0.35^**^ [0.29 to 0.40]	−0.36^**^ [−0.41 to −0.30]	−0.33^**^ [−0.39 to −0.27]	−0.03 [−0.09 to 0.03]	−0.37^**^ [−0.42 to −0.32]	−0.33^**^ [−0.39 to −0.27]	−0.51^**^ [−0.56 to −0.46]	-
M	0.20	4.82	5.04	5.13	4.36	4.77	2.94	5.55
SD	0.38	0.98	1.22	1.12	1.41	1.32	0.64	3.89
α	0.86	0.88	0.75	0.72	0.80	0.77	0.87	0.87

*N = 1,660*.

** *p < 0.01. SEA, Self-emotion appraisals; OEA, Others' emotion appraisals; ROE, Regulation of emotion; UOE, Use of emotion*.

### Multivariate statistical analysis

Multivariate statistical analysis was used to examine the influence of gender, age, grade levels and EI on self-esteem, suicide risk and cybervictimization. First, age was coded as a dummy variable (younger group = 0; older group = 1). In short, the younger group was made up with those adolescents between 12 and 14 years, and the older adolescent group was comprised of individuals aged between 15 and 18 years. Similarly, grade level was coded as a dummy variable: grades 7 and 8 were labeled as the lower grade levels (0), whereas grades 9–11 were classified as the higher grade levels (1). With respect to EI levels, the same procedure was followed (low *EI* = 0; high *EI* = 1). With the aim of dividing the sample into two groups, one with high and one with low EI levels, the adolescents who scored a standard deviation above the mean were included in the group with high EI, while the adolescents who had a standard deviation below the mean were included in the group with low EI. Therefore, gender, age, grade levels and EI levels were included as independent variables in order to test for its potential main and interaction effects.

The main effect for gender was significant [Wilks' λ = 0.97, *F*_(3, 484)_ = 5.93, *p* < 0.001, partial η2 = 0.035]. With respect to EI levels, similar results were found [Wilks' λ = 0.77, *F*_(3, 484)_ = 48.94, *p* < 0.001, partial η2 = 0.23]. However, no significant differences were found concerning age [Wilks' λ = 0.99, *F*_(3, 484)_ = 0.68, *p* = 0.57] nor grade level [Wilks' λ = 0.99, *F*_(3, 484)_ = 1.91, *p* = 0.13]. The interaction effects were not significant for gender x age [Wilks' λ = 0.99, *F*_(3, 484)_ = 0.31, *p* = 0.82], gender x grade [Wilks' λ = 0.99, *F*_(3, 484)_ = 2.07, *p* = 0.10], age x grade [Wilks' λ = 0.99, *F*_(3, 484)_ = 0.38, *p* = 0.77], gender x EI [Wilks' λ = 0.99, *F*_(3, 484)_ = 2.17, *p* = 0.09], age × EI [Wilks' λ = 0.99, *F*_(3, 484)_ = 0.50, *p* = 0.68] nor grade x EI [Wilks' λ = 0.99, *F*_(3, 484)_ = 2.35, *p* = 0.07].

In order to further test the overall gender and EI differences in cybervictimization, self-esteem and suicide-risk, several one-way ANOVAs were used. In addition, the effect sizes were calculated to describe the magnitude of gender and EI differences (Cohen, 1988). Effects sizes of 0.20, 0.50 and 0.80 were considered small, medium and large, respectively. Significant differences between boys and girls were found regarding the three indicators. In comparison to girls, boys reported lower cybervictimization [*t*_(1614)_ = −2.78, *p* < 0.010; *d* = −0.16], lower suicide risk [*t*_(1450)_ = −7.76, *p* < 0.001; *d* = −0.38] and higher self-esteem [*t*_(1614)_ = 5.75, *p* < 0.001; *d* = 0.28]. Similarly, significant differences were found between groups regarding low and high EI. Adolescents with low EI reported lower self-esteem [*t*_(500)_ = −18.40, *p* < 0.001; *d* = −1.63], higher cybervictimization [*t*_(350)_ = 4.52, *p* < 0.001; *d* = 0.40] and higher suicide risk [*t*_(500)_ = 11.61, *p* < 0.001; *d* = 1.01] in comparison to their high EI counterparts.

### Moderation analyses

We conducted moderation analyses to examine whether EI moderated the effect of cybervictimization on two indicators of psychological adjustment (self-esteem and suicide risk). Age, grade and gender were included as covariates in our models (steps 1, 2, and 3). Cybervictimization was entered in the fourth step as our independent variable, whereas the total score in EI was included in the fifth step. Lastly, the interaction of cybervictimization with EI was entered in the sixth step. Furthermore, to illustrate the effect of the interaction between cybervictimization and EI on self-esteem and suicide risk in adolescents, we plotted the regression following the procedures outlined by Hayes (2013).

With respect to self-esteem, a total of 28% of the variance was explained by the final model (see Table 2). First, we found that sociodemographic factors predicted 4% of the total variance. After controlling for these factors, cybervictimization was found to explain 4% of the variance in self-esteem scores. In addition, EI was found to account for an additional amount of self-esteem (20%) even after controlling the variance attributable to sociodemographic factors and cybervictimization. Finally, the cybervictimization x EI interaction explained a significant amount of the variance in self-esteem scores.

**Table 2:** Moderated hierarchical regression analyses for self-esteem and suicide risk.

	**R^2^**	**F**	**B**	**SE**	**β**	**95% CI**	**f^2^**	**ΔR^2^**
**SELF-ESTEEM**								
Step 1	0.020	33.40					0.02	0.020^***^
Gender			−0.18	0.03	−0.14^***^	−0.24 to −0.12		
Step 2	0.039	34.05					0.04	0.020^***^
Age			−0.06	0.01	−0.14^***^	−0.08 to −0.04		
Step 3	0.042	24.41					0.04	0.003^*^
Grade			0.05	0.02	0.10^*^	0.01 to 0.09		
Step 4	0.082	36.92					0.09	0.040^***^
Cybervictimization			−0.34	0.04	−0.20^***^	−0.42 to −0.26		
Step 5	0.279	128.30					0.39	0.198^***^
Emotional Intelligence			0.30	0.01	0.45^***^	0.27 to 0.32		
Step 6	0.282	108.00					0.39	0.002^*^
Cybervictimization × Emotional Intelligence			−0.03	0.01	−0.05^*^	−0.06 to −0.003		
**SUICIDE RISK**								
Step 1	0.035	59.89					0.04	0.035^***^
Gender			1.45	0.19	0.19^***^	1.09 to 1.82		
Step 2	0.041	35.27					0.04	0.006^***^
Age			0.20	0.06	0.08^***^	0.08 to 0.31		
Step 3	0.043	24.68					0.04	0.002
Grade			−0.24	0.13	−0.08	−0.49 to 0.02		
Step 4	0.154	75.28					0.18	0.111^***^
Cybervictimization			3.45	0.23	0.34^***^	2.99 to 3.91		
Step 5	0.240	104.21					0.32	0.086^***^
Emotional Intelligence			−1.19	0.09	−0.30^***^	−1.36 to −1.02		
Step 6	0.244	89.09					0.32	0.005^***^
Cybervictimization × Emotional Intelligence			−0.27	0.08	−0.07^***^	−0.44 to −0.11		

*B, Unstandardized beta; SE, Standard error of unstandardized beta; 95% CI = 95% Confidence Interval*.

* p < 0.05 and

*** *p < 0.001*.

**Figure 1:**
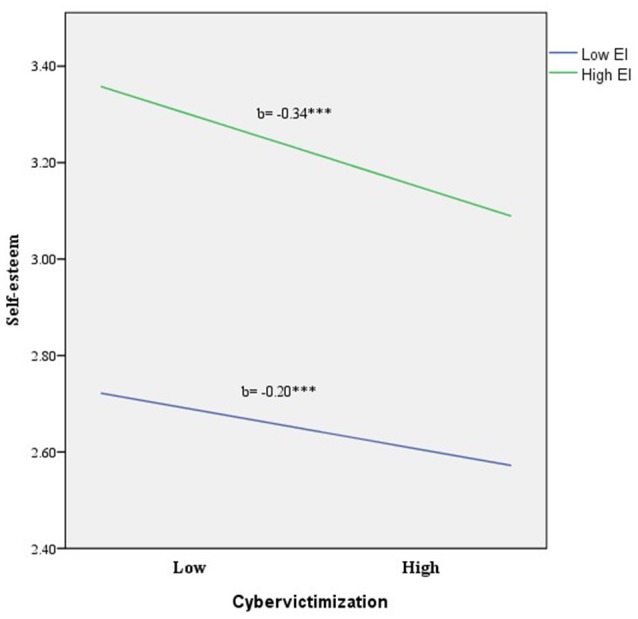
Relationship of cybervictimization and EI for predicting self-esteem, ^***^p < 0.001.

As can be seen in Figure 1, the relationship between cybervictimization and self-esteem weakened as EI increased. Specifically, the above-mentioned negative relationship between cybervictimization and self-esteem was significant at low levels of EI [*b* = −0.20, *t*_(1653)_ = −4.64, *p* < 0.001]. Quite interestingly, at higher levels of EI, the association between cybervictimization and self-esteem was also significant and even more intense [*b* = −0.34, *t*_(1653)_ = −6.02, *p* < 0.001].

Regarding suicide risk, a total of 24% of the variance in this variable was explained by the model (see Table 2). First, sociodemographic factors were found to predict 4% of the variance in suicide risk. In addition, cybervictimization was found to explain 11% of the variance in self-esteem scores even after controlling for these factors. Additionally, EI accounted for 9% of the variance in self-esteem, even after controlling the variance attributable to sociodemographic factors and cybervictimization. Lastly, we found that the cybervictimization x EI interaction explained a significant, unique component of variance in suicide risk.

**Figure 2:**
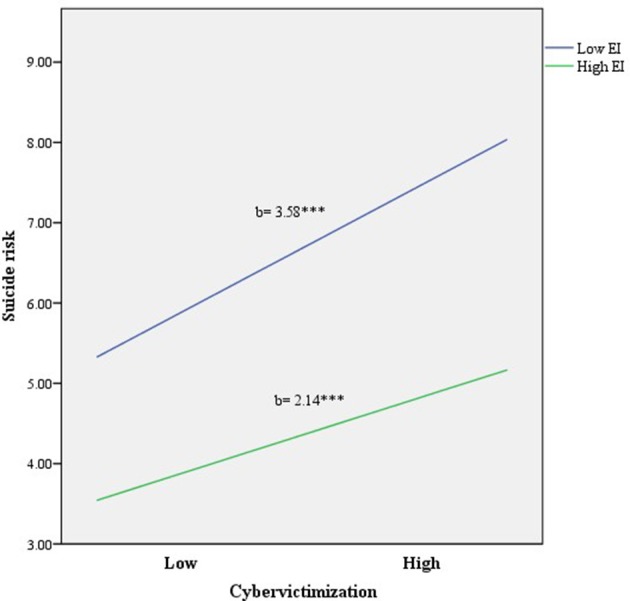
Relationship of cybervictimization and EI for predicting suicide risk, ^***^p < 0.001.

As Figure 2 shows, the association between cybervictimization and suicide risk weakened as EI increased. In particular, the positive association between cybervictimization and suicide risk was significant at low levels of EI [*b* = 3.58, *t*_(1653)_ = 13.40, *p* < 0.001]. At higher levels of EI, this relationship decreased although it remained significant [*b* = 2.14, *t*_(1653)_ = 5.80, *p* < 0.001].

## Discussion

Being a victim of cyberbullying has been linked to social, physical and psychological problems (Tokunaga, 2010; Palermiti et al., 2017). Although a meta-analytic study identified some key predictors of cyberbullying victimization by peers (Chen et al., 2017), more research is needed into factors that mitigate the negative consequences of cyberbullying victimization in adolescence. This study corroborates previous empirical research showing that cyberbullying (van Geel et al., 2014; Tsaousis, 2016) and EI (Elipe et al., 2015) influence mental health in adolescents. Our results also extend recent findings on the role of emotional abilities in buffering the negative consequences of cyberbullying victimization (Baroncelli and Ciucci, 2014; Hemphill et al., 2014), as they show that EI may also moderate the association between cyberbullying victimization and psychological maladjustment, operationalized as low self-esteem and high suicidal ideation.

Regarding our hypothesis on the expected relationship between variables, in line with prior work, we found that cyberbullying victimization was positively associated with suicide risk (van Geel et al., 2014) and negatively related to self-esteem (Tsaousis, 2016). These results confirm earlier research suggesting that being a victim of cyberbullying has a negative impact on both physical and psychological health (Takizawa et al., 2014; Tsaousis, 2016; Palermiti et al., 2017). In general, individuals have a fundamental psychological need to belong to a peer group and to be accepted by their peers (Baumeister and Leary, 1995). Therefore, being cyberbullied in adolescence may cause psychological maladjustment and reduced well-being (Parker et al., 2006). Our results also suggest that being cyberbullied has a considerable impact on the development of negative emotional responses that can lead to reduced levels of psychological adjustment such as increased suicidal ideation and behaviors and decreased self-esteem (Mishna et al., 2011; Campbell et al., 2012). Moreover, in line with previous research, the results showed that girls reported higher levels of cybervictimization (Craig et al., 2009), as well as more adverse psychological maladjustment compared to boys (e.g., Zych et al., 2015). One plausible explanation might be related to the definition of cybervictimization, which could be understood as an indirect type of bullying who often girls are more likely to experience (Beckman et al., 2013). However, further studies are needed to provide a more nuanced picture of gender and other sociodemographic differences in mental health indicators (Zych et al., 2015).

Regarding our hypothesis about the buffering role of EI in the cybervictimization-mental health link, drawing on prior meta-analytical research (Chen et al., 2017), our results indicate that EI might also be a personal resource that might alleviate the negative psychological symptoms in adolescents at risk of cybervictimization. Earlier work has suggested that EI can buffer against the negative effects of stressful life events on self-reported mental health (Davis and Humphrey, 2012a), our results extend this finding by showing that cyberbullying is associated with increased suicidal ideation and low self-esteem at all levels of EI, which indicates that cyberbullying has a severely deleterious effects on the health of its victims. Adolescent victims of cyberbullying may experience negative psychological symptoms as a result of repeated cyberaggression against which they are unable to defend themselves. However, independently of cyberbullying victimization, adolescents with greater EI were less likely to report symptoms of suicidal ideation and low self-esteem than their lower EI peers. It is, therefore, possible that cyberbullying has less impact on the suicidality of adolescent victims and self-esteem if they have high EI. Possessing the emotional abilities grouped together as EI - perception of emotions, understanding of the causes and consequences of emotions and the ability to manage the emotions of oneself and others-may reduce the risk that adolescent victims of cyberbullying might experience psychological problems as a consequence.

## Limitations and future research

Our study presents several limitations, which provide an avenue for further research. We used a self-report measure of EI, but future studies should use EI performance tests such as the Mayer-Salovey-Caruso Emotional Intelligence Test (Mayer et al., 2003) in order to generalize our findings. In addition, although the ordering of variables in our analyses was grounded in theoretical work on EI and cyberbullying victimization (Elipe et al., 2015) and cyberbullying victimization and psychological problems (van Geel et al., 2014; Tsaousis, 2016) the cross-sectional nature of the data means it is impossible to determine causality. Replicating our findings in a longitudinal study in which EI is measured at the start of an academic course and cyberbullying victimization and negative psychological symptoms are measured at a later date would provide further insights to the causal relationships among EI, cyberbullying victimization and mental health. It is also important to underline that our adolescent participants were healthy and that our findings may not generalize to victims of cyberbullying diagnosed with post-traumatic stress disorder. Also, our study used pencil and paper. Further studies, especially in the field of cyberbullying, might be conducted online which would save time in measuring a large sample and some bias might be reduced. Relatedly, future studies should examine differential profiles of personal resources such as EI regarding experiences of cybervictimization. Finally, further research is needed to examine the potential buffering role of EI considering specific samples of cybervictimized adolescents.

### Theoretical and practical implications

These limitations notwithstanding, there are several implications of these findings for research and practice. Theoretically, our findings suggest that being a victim of cyberbullying has a greater effect on adolescents' self-esteem and suicidal ideation. Such effects might also be more negative if victims do not believe that their emotional resources are adequate for coping with being cyberbullied. It is, therefore, plausible that stressful experiences such as being cyberbullied might have a cumulative negative impact on young people, leading to low self-esteem and high suicidal ideation. However, one's level of EI may influence how one interprets and reacts to cyberbullying, thus high EI may mitigate some of the negative consequences (Elipe et al., 2015). In other words, while cyberbullying victimization may be a risk factor for negative psychological symptoms in adolescence, EI could be an important moderator of this association. Although theoretical models of cyberbullying have consistently documented the detrimental effect on mental health (Mishna et al., 2011; Campbell et al., 2012), identifying the protective factors (i.e., EI) that shield victims from the adverse health consequences of cyberbullying is an important avenue and might offer a more comprehensive theoretical framework. Regarding practice, prevention and intervention programs should incorporate not only a whole-school anti-bullying policy and curriculum-based activities to prevent cyberaggression, but also a variety of EI-based strategies to reduce the adverse symptoms associated with being cyberbullied by peers. Cyberbullying prevention programs are currently available online, so parents and caregivers should use the websites associated with such programs as a source of information about how to discuss cyberbullying with young people. Moreover, school practitioners might draw on these websites to teach students how to identify electronic forms of aggression, to recognize potential psychosocial symptoms of cyberbullying victimization, to resolve conflicts and to use nonviolent problem-solving techniques in order to increase the personal resources at their disposal and thus reduce the likelihood of psychological maladjustment or interpersonal dysfunction arising in adolescence. Accordingly, since electronic forms of adolescent aggression have been shown to be linked to psychological problems, and may indirectly influence the learning environment at school (Zimmerman et al., 1997), educators and school psychology practitioners should make preventing these forms of aggression a priority, even when the incidents occur off-campus. For example, parents should be contacted and students should be disciplined if their behavior threatens the academic and psychological outcomes of other students. Thus, the EI of adolescents is another potential intervention target for initiatives to reduce the incidence and impact of cyberbullying. School practitioners might be able to mitigate some of the negative consequences of cyberbullying by developing the EI of potential adolescent victims so that they are better able to manage negative emotions such as worry, fear, helplessness and anxiety. Similarly, EI training might make adolescents more resilient to the negative effectives of electronic aggression on self-esteem by helping to cognitively restructure how it is perceived and increasing their awareness of the emotional impact of hurtful messages on self-concept and giving them better strategies for coping with cyberbullying. Improving the EI of adolescents may not only help victims of cyberbullying to cope, but also it may also help bystanders and perpetrators to recognize cyberbullying, to understand its emotional impact on victims and the importance of preventing it from happening. As we found that EI acted as a buffer against the negative impact of cyberbullying on suicidal ideation and self-esteem, future research could include investigating whether teaching adolescents EI skills reduces the psychological symptoms associated with being cyberbullied by peers and changes the status of victims in their peer group. EI interventions might also have a positive impact on school climate and interpersonal relationships between peers (Durlak et al., 2011), academic performance (Perera and DiGiacomo, 2013) and wellbeing (Sánchez-Álvarez et al., 2016) which are key factors for school success.

## Conclusion

Despite these limitations, our research provides further empirical evidence that EI should be considered as a personal resource that is relevant to the negative symptoms associated with cyberbullying victimization. Our findings contribute to the theoretical literature on cyberbullying and its negative consequences in adolescence, but they could also be used to develop school-based, integrated bullying prevention programs aimed at increasing the emotional abilities of adolescents in order to protect against, or at least mitigate, the negative consequences of being a victim of cyberbullying.

## Ethics statement

This study was carried out in accordance with the recommendations of Ethics Committee of the University of Málaga (Spain), with informed consent from all participants. Directors' institute gave informed consent in accordance with the Declaration of Helsinki. The protocol was approved by Research Ethics Committee of the University of Malaga (62-2016-H).

## Author contributions

All authors participated and contributed in study design, data collection, statistical analysis, interpretation of data, and drafted the manuscript. Besides, all authors read and approved the final manuscript.

### Conflict of interest statement

The authors declare that the research was conducted in the absence of any commercial or financial relationships that could be construed as a potential conflict of interest.
